# Trade-Offs Between Simplifying Inertial Measurement Unit–Based Movement Recordings and the Attainability of Different Levels of Analyses: Systematic Assessment of Method Variations

**DOI:** 10.2196/58078

**Published:** 2025-06-03

**Authors:** Manu Airaksinen, Okko Räsänen, Sampsa Vanhatalo

**Affiliations:** 1 BABA Center, University of Helsinki and Helsinki University Hospital, Haartmaninkatu 8, Helsinki, 00290, Finland, 358 50 3072439; 2 Signal Processing Research Centre, Tampere University, Tampere, Finland; 3 Department of Physiology, University of Helsinki, Helsinki, Finland

**Keywords:** human activity recognition, recording configuration, wearable, movement sensors, gross motor development, neurodevelopment, neural networks, MAIJU, IMU, sensor, algorithm, detection, balance, motility, activity recognition, motor, motor development, posture, motility assessment, accelerometer

## Abstract

**Background:**

Human movement activity is commonly recorded with inertial measurement unit (IMU) sensors in many science disciplines. The IMU data can be used for an algorithmic detection of different postures and movements, which may support more detailed assessments of complex behaviors, such as daily activities. Studies on human behavior in real-life environments need to strike a balance between simplifying the recording settings and preserving sufficient analytic gains. It is poorly understood, however, what the trade-offs are between alternative recording configurations and the attainable analyses of naturalistic behavior at different levels of inspection, or with respect to achievable scientific questions.

**Objective:**

This study assessed systematically the effects of IMU recording configurations (placement and number of IMU sensors, sampling frequency, and sensor modality) on the high temporal resolution detections of postures and movements, and on their lower temporal resolution derivative statistics when the data represents naturalistic daily activity without excessively repetitive movements.

**Methods:**

We used a dataset from spontaneously moving infants (N=41; age range 4‐18 months) recorded with a multisensor wearable suit. The analysis benchmark was obtained using human annotations of postures and movements from a synchronously recorded video, and the reference IMU recording configuration included 4 IMU sensors collecting triaxial accelerometer and gyroscope modalities at 52 Hz. Then, we systematically tested how the algorithmic classification of postures (N=7), and movements (N=9), as well as their distributions and a derivative motor performance score, are affected by reducing IMU data sampling frequency, sensor modality, and sensor placement.

**Results:**

Our results show that reducing the number of sensors has a significant effect on classifier performance, and the single sensor configurations were nonfeasible (posture classification Cohen kappa<0.75; movement<0.45). Reducing sensor modalities to accelerometer only, that is, dropping gyroscope data, leads to a modest reduction in movement classification performance (kappa=0.50-0.53). However, the sampling frequency could be reduced from 52 to 6 Hz with negligible effects on the classifications (posture kappa=0.90-0.92; movement=0.56-0.58).

**Conclusions:**

The present findings highlight the significant trade-offs between IMU recording configurations and the attainability of sufficiently reliable analyses at different levels. Notably, the single-sensor recordings employed in most of the literature and wearable solutions are of very limited use when assessing the key aspects of real-world movement behavior at relevant temporal resolutions. The minimal configuration with an acceptable classifier performance includes at least a combination of one upper and one lower extremity sensor, at least 13 Hz sampling frequency, and at least an accelerometer, but preferably also a gyroscope (posture kappa=0.89-0.91; movement=0.50-0.53). These findings have direct implications for the design of future studies and wearable solutions that aim to quantify spontaneously occurring postures and movements in natural behaviors.

## Introduction

There is a wide interest in studying human activity with inertial measurement unit (IMU)-sensors in a variety of disciplines, including health care, developmental psychology, education, and sociology [1-6]. The objective physical measures of acceleration and gyration from the IMU sensors are considered to provide a significant complement, or alternative, to the traditionally used questionnaires [7] and expert assessments [8], both of which are compromised by subjectiveness and inherent ambiguities. Moreover, the expert-based assessments in the lab or hospital setting are costly and labor-intensive, whereas IMU sensors can be used as wearables to measure human activity in the real world, such as in participants’ homes, supporting substantially improved ecological validity of the findings.

Compared with the many other methods used in movement studies, such as surface electromyography, video recordings, or 3D motion capture (3D movement analysis), the currently available IMU sensors allow for an easy-to-use [9], cost-efficient, privacy-preserving [10], and domain-relevant collection of movement data that can be easily deployed for long-form recording. IMU-sensor recordings are well accepted by the participants and their caregivers (in the case of minors), and they are relatively easy to use in long-term naturalistic measurements [11,12]. Moreover, the analyses and interpretability of IMU signals may be considerably easier than analyzing electromyography signals or video recordings obtained in uncontrolled environments.

The traditional approach to IMU-sensor data collection is to measure accelerometry (“actigraphy”) signals from a single wrist-, hip, or ankle-worn accelerometer sensor [13], followed by quantifying the amounts of movements as total, with break-down to different levels of activity (eg sedentary, light, or moderate to vigorous), or to indirectly estimate the related energy expenditure [1,14-16]. The single-sensor setups are used in many commercially available wellness products to categorize sustained and repetitive activities (eg, sitting, lying, walking, running, climbing, and cycling [17,18]). Further, the recently introduced parallel gyroscope modality has increased its accuracy [19-21]. This methodologically simple approach reduces movement analysis to amounts of movements or detections of repetitive activities only, both of which fail to account for the frequently changing postures and movements, the key components of all natural human behavior. At the other extreme of recording setups, a very high number of IMU sensors may support detailed skeletal pose modeling [22-24]. However, the technical and analytic complexity of such measurements has limited their use to dedicated laboratory environments only.

An intermediate form between these extremes would be multi-IMU recording setups with a handful (N=2‐6) of sensors, which have been used increasingly to improve the analytic range of human activity recognition (HAR); however, studies with such setups have used acted-out data collection with predefined activity types (eg, [18,25,26]), which inherently limits their generalizability to real-life measurements [27]. For at-home measurements of naturalistic activity, we have recently developed a multisensor wearable suit for infants (MAIJU, motor ability assessment of infants with a jumpsuit [28-32]), using 4 IMU sensors that collect triaxial accelerometer and gyroscope signals at 52 Hz. The data analysis uses a validated, deep learning–based analytical pipeline to provide a human-equivalent level classification of postures (7 categories) and generic movements (9 categories) for each second of the recording [29]. These high-temporal-resolution/low-analytic-level detections can be used further to build low-temporal-resolution/high-analytic-level assessments of daily activity or motor development [29,30].

In order to maximize the scalability and utility of the novel IMU technology, it would be essential to know the exact trade-offs between simplifying the recording setup and the losses of achievable analyses at different levels of inspection. For example, out-of-lab recordings would benefit from a lower number of sensors that simplify the wearable gear design, or lower data rates that increase the maximum recording lengths and reduce errors in data logistics; meanwhile, well-controlled environments with brief recordings would benefit from redundancy in the setup that increases analytical accuracy. Prior studies have assessed selected aspects of these trade-offs primarily on acted-out or otherwise controlled (ie, nonspontaneous) activities. They have reported that at least 2 sensors with either thigh and ankle [6] or thigh and shoulder [28] positioning are required to achieve adequate posture tracking with a high temporal resolution, and the sampling rate is likely not a significant analytic bottleneck between 10 and 100Hz [18,26,33].

Here, we studied systematically how the recording configurations in naturalistic measurements would affect the accuracy of analytic details at different levels of inspection, ranging from high-temporal-resolution and low-analytic-level detections to low-temporal-resolution and high-analytic-level assessments of spontaneous behavior. The overarching aim was to define which sensor placements, sampling frequencies, and sensor modalities are necessary for different levels of study questions. We used a multisensor IMU dataset with second-by-second level expert annotations of well-defined movements and postures in naturalistic settings, which together provide a rich, previously published and fully generalizable (ie, age-independent) presentation of spontaneously occurring movement behaviors. The novel contribution of this work is the systematic evaluation of the effects of (1) the number of sensors over 4 specific proximal limb locations, (2) sensor modality, and (3) sampling frequency, in the area of classifying posture and movement and their higher-order aggregate and derivative summary metrics for 4‐18-month-old infants.

## Methods

### Overview

The overall goal was to study the effect of the recording configuration on the automated, quantified movement and posture analyses. The classifier outputs were assessed at high time resolution using previously developed second-by-second outputs [28,29], followed by assessing summary representations over the recording session (category distributions and scalar projections [29,30]). The ground truth of postures and movements was taken from the human inter-rater agreement obtained from independent annotations using IMU-synchronized video recordings.

### Data Collection and Recordings

#### MAIJU Wearable

**Figure 1.**
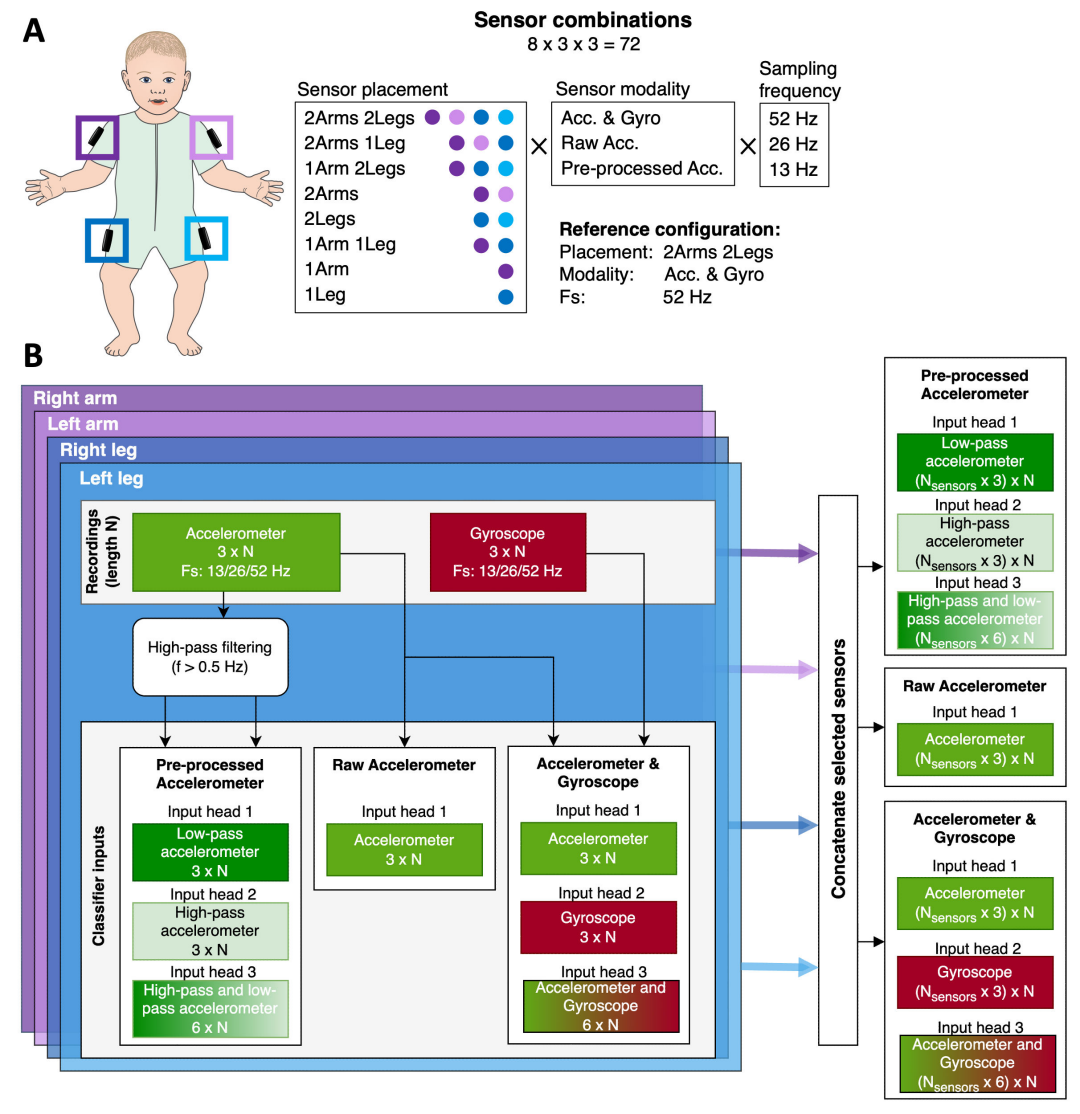
Overview of the study. (**A**) Illustration of the studied recording configurations that are obtained by subsampling from the reference configuration data. (**B**) Block diagram depicting the data preprocessing for each configuration. The “raw accelerometer” and “accelerometer+gyroscope” configurations take in the raw inertial measurement unit (IMU) signals (gyroscope with bias removal), whereas the “pre-processed accelerometer” configuration preprocesses the raw accelerometer signal into low- and high-pass parts, after which they are treated as independent data streams.

MAIJU (Figure 1 [28-30]) is a full-body overall akin to a commonplace infant swimsuit that is equipped with 4 movement sensors (Movesense [34]), one proximally placed in each limb. The sensors stream IMU data via a Bluetooth 5.0 low-energy connection into an iOS-based mobile device with a custom-built application, “Maiju logger” (Kaasa GmbH, Düsseldorf, Germany). Data collection is performed with a 52 Hz sampling rate using a triaxial accelerometer (measuring linear acceleration at m/s^2^; sensitivity 8 g) and gyroscope (measuring angular velocity at deg/s; sensitivity ±500 deg/s).

#### IMU Recording Configurations

Prior work with the MAIJU recordings has been published with the highest available configuration setup (4 sensors; accelerometer and gyroscope; 52 Hz sampling). This reference configuration was shown to enable classifier performance at a human equivalent level, ie, the agreement between the classifier and a human expert is comparable to human inter-rater agreement [28,29]. Our previous experiments have shown that classifier robustness can be improved with simulated sensor dropout [31], and other studies on infants have shown that 2 or 3 IMU sensors may provide successful posture classification [4], if the sensors are attached to the unilateral hip, ankle, and waist. Initial experiments with MAIJU demonstrated that an incomplete list of postures is detectable with 2 sensors [28]. These results together suggest that the number of IMU sensors could be reduced from 4. Regarding the sensor locations, the proximal thigh placement (laterally in MAIJU wearable) is identified as the most robust single sensor location for reliable capture of different human activities [17].

In this work, we studied systematically how reductions in recording configurations affect the movement analyses at different levels of inspection, ranging from high-resolution (1 s level) classifications to summary measures of the whole recording session. The tested 3 domains of varying recording configurations were (Figure 1): sensor placement, sensor modality, and sampling frequency. Sensor placement refers to the number of sensors used (from 1 to 4) at the different (proximal-lateral-) limb locations. The recording modality of the sensors can be selected as either an accelerometer, a gyroscope, or both. Since the gyroscope requires orders of magnitude more power compared with the accelerometer, and it does not contain information about the absolute state of orientation, we omitted the gyroscope-only configuration from the study. Since we are using an end-to-end neural network for our classifier, we tested 2 variants of accelerometer-only configurations to reflect the 2 distinct physical sources of acceleration—the gravity component (limb orientation) and limb acceleration—which were obtained by splitting the accelerometer signal into low-pass and high-pass components, respectively.

The “raw accelerometer” variant uses solely the measured accelerometer as such, and in the configuration “pre-processed accelerometer” the low- and high-pass filtered components are fed into separate input heads (Figure 1B). Finally, we test all of the configuration variants with 3 different sampling frequencies supported by the Movesense sensor (52, 26, and 13 Hz), all of which are obtained from the original reference configuration recording by decimation followed by upsampling and low-pass filtering.

#### Dataset Details

This study uses 29.3 hours (91,449 frames) of previously published data [29] that includes 41 recordings from infants aged 4.5‐16.6 months. The data, alongside parallel video recordings, were obtained at the infants’ homes (N=17) or at the lab within a home-like setting (N=24) during minimally structured play sessions that lasted from 18 minutes to 74 minutes.

#### Annotations

**Figure 2.**
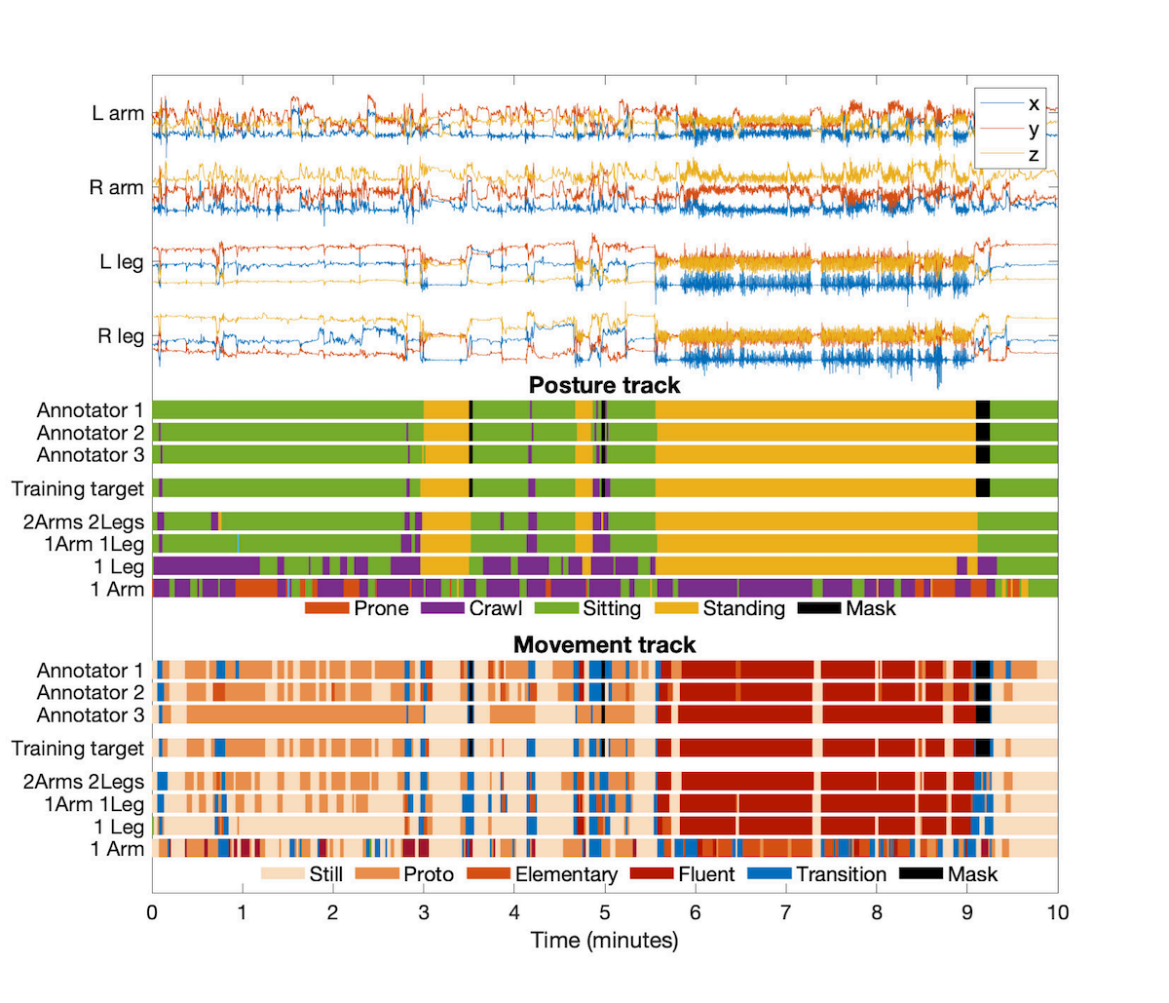
Example of the raw accelerometer signals and the video-based annotations for posture and movement within a 10-minute recording segment. For each track, the 3 parallel annotations are depicted alongside the training target (obtained with iterative annotation refinement [28]). The classifier outputs are presented with 4 different configurations: 2Arms 2Legs (52 Hz, acc and gyro), 1Arm 1Leg (13 Hz, pre-processed acc), 1 Leg (13 Hz, pre-processed acc), and 1 Arm (13 Hz, pre-processed acc). Note the increasing classification errors when reducing the recording configurations, and the apparently random classification with 1 arm recording.

**Figure 3.**
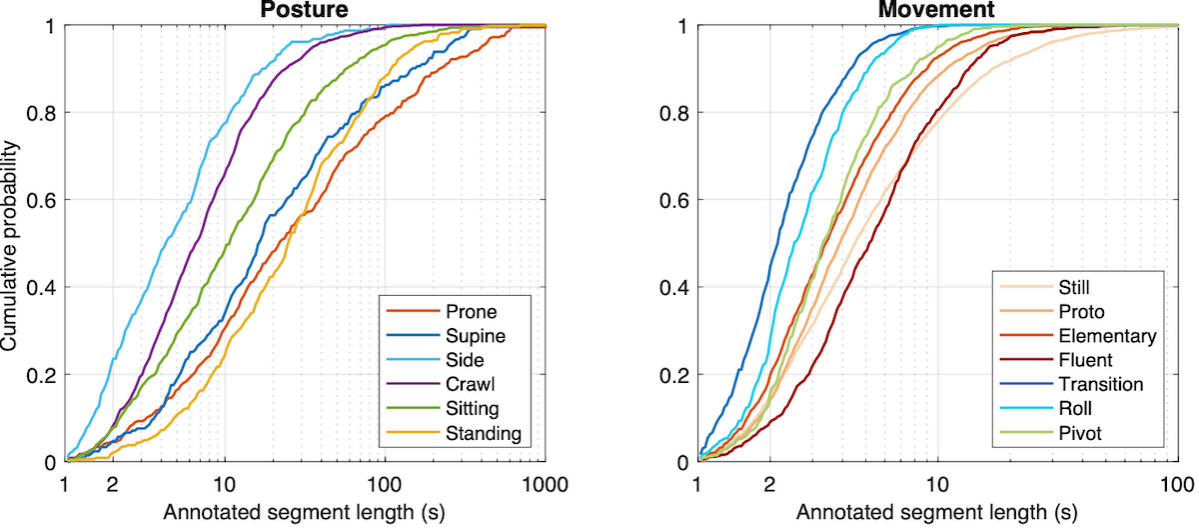
Cumulative distributions for the number of posture and movement categories as a function of segment length.

We used a previously reported set of human annotations [29] from 3 (N=34) or 2 (N=9) independent annotators. The annotations were performed with the free Anvil software [35], which presents synchronized video and raw signal waveforms to annotate 3 separate tracks: posture, movement, and auxiliary (for details, see [29]). The posture track contains 7 categories (prone, supine, side left, side right, crawl posture, sitting, and standing), and the movement track contains 9 categories (still, proto, elementary, fluent, transition, roll left, roll right, pivot left, and pivot right) (Figure 2). The auxiliary track contained segments where posture and movement annotation could not be performed, viz, infant being carried instead of independent movement and out-of-camera segments. These segments were not included in training or evaluation and have not been counted in the reported dataset size. The baseline Cohen kappa scores for the multiclass inter-rater agreement for the posture and movement annotations were 0.93 (95% CI 0.92 to 0.94) and 0.59 (95% CI 0.57 to 0.62), respectively. As discussed in detail in [28,29], the posture and movement tracks are designed to represent complementary aspects of human movement behavior, and due to the differences in their conceptual clarity and temporal dynamics (Figure 3), the kappa levels between the tracks should not be directly compared. Specifically, the accuracy metrics of the movement track suffer from short bout lengths because the true category transitions cannot be perfectly aligned with the analytic time frames. This unavoidable compromise in temporal accuracy arises from real-world behavior. Notably, our prior work has demonstrated how it is effectively mitigated by using aggregate statistics over the measurement session, yielding unbiased movement track accuracies that are highly comparable with human visual annotations [29] and that have direct utility in describing the gross motor developmental level [36]. Further, our prior work has demonstrated that the movement categories can be meaningfully combined, for example, to denote a dichotomy between “locomotion” and “non-locomotion” [37].

To estimate suitable window lengths for short-term analysis, we studied durations of the posture and movement segments in the data for different categories. In principle, the choice of window length is a compromise of 2 factors: more data in the longer windows would typically improve classifications, however, a too-long window length leads to contamination by different activity types inside the same window. The cumulative distributions for the counts of variable-length annotation segments are presented in Figure 3. The figure shows that almost none of the annotations are less than 1 second in length, whereas especially for movement, there are substantial amounts of segments with duration <2 seconds (≈10%‐40%, depending on the movement category; corresponding roughly to ≈5%‐20% of total time). This suggests that the length of analysis windows should be within 2 seconds to capture all annotated activities. Thus, we used the previously published [28-32], window length of 2.3 seconds (120 samples at 52 Hz) with 50% overlap (1.15 s; 60 samples at 52 Hz).

### Data Preprocessing and Neural Network-Based Classifier

#### Data Preprocessing

##### Windowing

**Figure 4.**
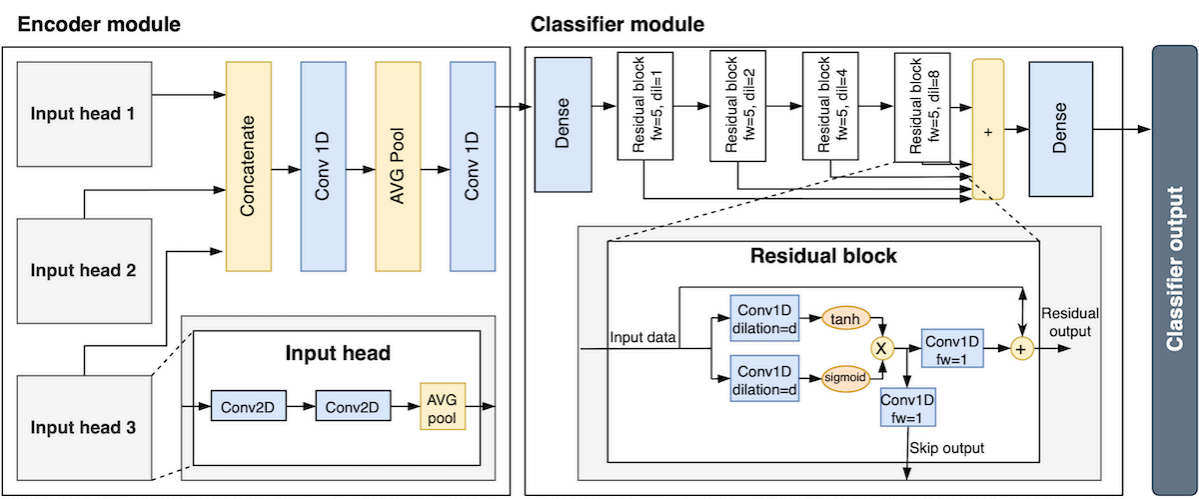
Block diagram depicting the neural network structure for all of the classifiers.

The raw IMU-sensor data is segmented into 2.3 s (120 samples per channel) frames with 50% overlap. The frames are processed and concatenated based on modality (Figure 1B) and then fed into the classifier (Figure 4).

##### Downsampling

The used Movesense sensor firmware supports measurement at sampling rates starting from 13 Hz. We simulated sampling frequencies lower than 52 Hz (reference configuration) by decimating the original signal by a factor of 2 or 4 (for 26 Hz and 13 Hz, respectively) and then upsampling by the same factor followed by forward-backward low-pass filtering with a sixth order Butterworth filter (with a cut-off frequency of 0.5 * decimated sampling frequency). This was done to keep the classifier architectures identical (ie, each variant takes in frames of 120 samples).

##### Pre-Processed Accelerometer

In the pre-processed accelerometer modality, the obtained raw accelerometer signal is split into low-pass and high-pass components that are processed in parallel blocks (Figure 4). The low-pass component in accelerometers is primarily caused by the earth’s gravity, and it contains information about the absolute orientation of the sensor, whereas the high-pass component primarily contains information about the instantaneous movement. The pre-processed accelerometer signals were obtained by forward-backward filtering the raw accelerometer signals with an eighth-order Butterworth filter with a cut-off frequency of 0.5 Hz (for the low-pass component). The high-pass component was obtained by subtracting the low-pass component from the original signal.

### Classifier Architecture

The overall architecture of the end-to-end neural network classifier is presented in Figure 4, and is identical to our earlier experiments [28-32]. The architecture consists of an encoder module (responsible for condensing frame-level information) and a classification module (which performs time-series modeling over the frame-level features). The encoder module has a varying amount of input heads, depending on the chosen modality (1 for raw accelerometer, 3 for the rest). Each input head has a similar architecture but takes as input the concatenated channels of the different sensor modalities (Figure 1B). The modality-level information is combined within the input head to latent representations that are concatenated and further processed in the final layers of the encoder module to obtain a 160-dimensional latent representation for each frame. The classifier module contains stacked residual blocks that do dilated convolutions over the frame-level representations of the data tensor (looking symmetrically into the past and future with a total receptive field of 30 frames=34.6 s).

### Training Details

All classifier training experiments were performed with recording-level 10-fold cross-validation with a fixed random seed. Minibatch gradient descent with the ADAM optimization algorithm was used with a weighted categorical cross-entropy loss (batch size=1×100 consecutive frames, learning rate 10^−4^, beta_1_=.9, beta_2_=.999, epsilon=10^−8^). To mitigate the effects of unbalanced category distributions, each frame’s error in the loss functions was weighted with the inverse probability of the target class occurrence. Sensor dropout (*P*=.3), sample dropout (*P*=.3), and random Euler rotation (*P*=.3, ±15 deg) were applied randomly during training to the input signals to enhance model robustness. The training was run for 200 epochs, and held-out validation data (20% of training data) was used to select the best-performing model in terms of the Cohen kappa score.

### Levels of Analysis Outputs

#### Overview

The classifiers for posture and movement produce frame-level information at a rate of 0.87 frames/s. Many use cases, such as health care or developmental research, are interested in holistic estimates of overall movement performance, and they need summary metrics from the whole recording with a fixed-length feature vector or a scalar value representing the recording. In this study, we used 2 such summary metrics at the level of individual infants: concatenated category distribution vectors and a holistic scalar value, BABA infant motor score (BIMS) [29].

#### Recording-Level Category Distributions

The classifiers produce a one-hot coded vector (1, N_cats_) for each frame, where N_cats_ is the number of output categories in the classifier. Thus, for each track in a recording, the classifiers output a matrix of shape (N_frames_, N_cats_), where N_frames_ is the number of frames in the recording (at 1.15 s intervals). An automatic carrying detection classifier [32] produces a binary mask of shape (N_frames_, 1), which is applied as a filter to the posture and movement classifier outputs before computing the distributions. The distribution for each track is computed by taking the mean of the automatic carrying detection-filtered one-hot matrices along the frame axis to yield a vector of shape (1, N_cats_). It is possible to combine the posture and movement classifications into a single “posture-conditioned movement” representation (ie, prone-still, prone-fluent, standing-still, etc), which yields a total of 27 sensible combinations of motor activity (ie, standing-fluent is a sensible combination, but supine-fluent is not). After obtaining the recording-level distribution value p’_c(m_i)_, where *c* is the category in question, *i* is the recording number, and *m* is the MAIJU recording, the values were concatenated across all recordings into a vector p’=[p’_c(m_i)_] and compared with the annotation-based reference values [p_c(m_i)_] with Pearson *r*. 95% CIs were obtained with bootstrapping (10,000 iterations) at the recording level.

#### Recording-Level Holistic Assessment

One of the targets of MAIJU recordings is to track the development of gross motor abilities. A novel index BIMS was introduced [29] as a holistic scalar measure for gross motor maturity. BIMS is a normalized (to a scale of [0, 100]), unitless measure defined as the statistical expectation of age that has most likely generated the given measurement. The concatenated recording-level posture and posture-conditioned movement distributions are used as feature vectors within Gaussian process regression [38] for the BIMS computation [30]. Our previous experiments have shown that BIMS correlates well with a conventional assessment battery of gross motor ability (Alberta Infant Motor Scale) [8].

### Performance Metrics

Performance for frame-level classification is computed from a compounded confusion matrix where each individual human annotation is compared with the classifier output. For inter-rater agreement, the individual annotations are compared with each other in all combinations. The confusion matrices over all recordings are summed into a single compound confusion matrix, from which the number of true positives (tp), true negatives (tn), false positives (fp), and false negatives (fn) are obtained. Overall performance is reported as Cohen kappa with the formula:


$$k=\frac{2(tp^{*}tn-fn^{*}fp)}{\left(tp+fp)(fp+tn)+(tp+fn)(fn+tn\right)}$$

Due to the unbalanced nature of the category distributions, kappa gives a more informative (compared with accuracy) correlation-like score (in [–1,1]), where a score of 0 denotes chance-level performance.

### Statistical Methods

#### Paired *t* Test

Two-tailed paired *t* tests are used to test for the null hypothesis that the differences between otherwise equivalent systems’ performances are zero when accounting for a single configuration variable. The results are reported with significance levels of (*P<.05; **P<.001; ***P<.0001). To justify the use of the *t* test, the Gaussianity of the differences is verified with a single-sample Kolmogorov-Smirnov test with *P*>.05.

#### Pearson *r*

The aggregate metrics are compared between the annotation-based and classifier-based variants with the Pearson correlation coefficient. This is due to the naturally uneven distribution of category occurrence, which makes straightforward loss metrics such as L1 or L2 loss hard to interpret. Aggregate scores include the category distributions and the BIMS score. The use of Pearson *r* is justified by a strong linear dependency between the tested variables.

#### Bootstrapping

The basic bootstrapping method is used to obtain 95% CIs for the accuracy, Cohen kappa, and Pearson *r* values. In each case, the sampling (10,000 iterations) is performed at the recording level when computing the compounded result. The *t* test is not used in connection with bootstrapping, where statistical significance is interpreted in terms of the overlap of the CIs.

### Ethical Considerations

The study complies with the Declaration of Helsinki, and the research was approved by the Ethics Committee of Children’s Hospital (number HUS-1331-2016-8; September 14, 2015), Helsinki University Hospital. A written informed consent was obtained from one of the parents of each participant.

## Results

### Overview

We present the results of 3 experiments to assess the effects of reducing recording configurations (placement, amount, modality, sampling frequency) to (1) high-resolution classification of posture and movement (1.15 frames per second), (2) to recording-level summary measures, and (3) to holistic assessment on infants’ gross motor development. For all experiments, the classifier performance was assessed with recording-level 10-fold cross-validation.

### Experiment 1: Effect of Reducing Recording Configuration to High-Resolution Classification

#### Overview

We trained separate posture/movement classifiers for all reduced IMU sensor inputs to study how the classifier performance degrades as a function of compromising the IMU configurations. A total number of (8x3×3) 72 classifiers for both tasks (Figure 1) were trained with varying recording configurations, including 8 variants of sensor placement, 3 sampling frequencies (52, 26, and 13 Hz), and 3 alternatives of measurement modality (acc and gyro, raw acc, pre-processed acc) (see Methods section).

#### Sensor Placement

**Figure 5.**
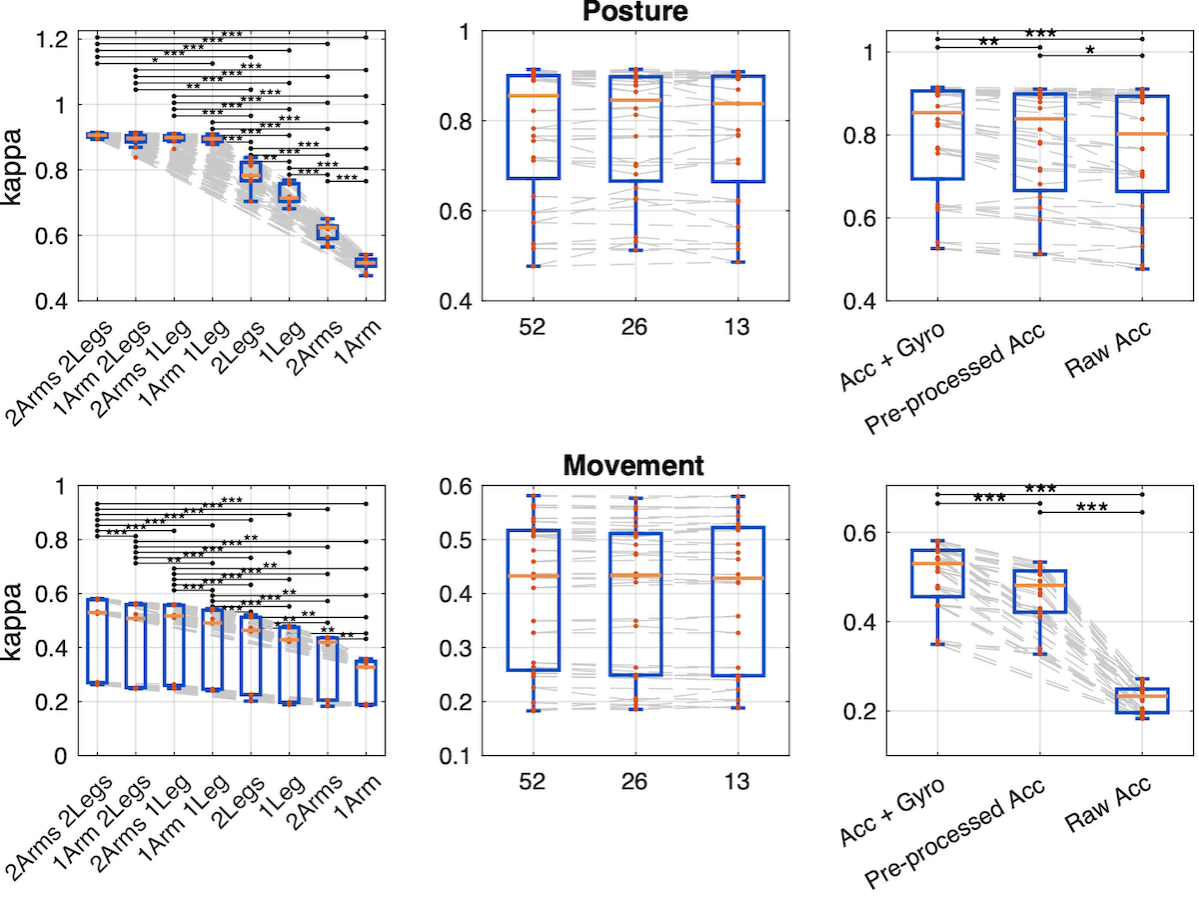
Effect of recording configuration to frame-level classification. Effect of sensor placement (left), sampling frequency (middle), and sensor modality (right) on the classification performance (Cohen kappa) for posture (top) and movement (bottom). The boxplots show the median, IQR, and range of experiment values for the cases where the given attribute is held constant. The gray lines connect systems with otherwise equivalent parameters. Two-tailed paired *t* tests are used to test for the null hypothesis that the differences between otherwise equivalent systems’ performances are zero (*P<.05; **P<.005; ***P<.001). Please note the different y-axes for posture and movement to support within-task comparisons across configurations.

The full classifier performance results are shown in Figures5  6, ordered by descending order of mean kappa levels. Notably, the same rank hierarchy of recording configurations emerges for both posture and movement detections: 2Arm2Leg > 1Arm2Leg > 2Arm1Leg > 1Arm1Leg > 2Leg > 1Leg > 2Arm > 1Arm. For postures, however, the 3 best-performing configurations are not significantly different (*t*_8_ range 0.4‐2; *P*=.08-.59). These results together indicate clearly that a combination of upper and lower limb IMU sensors yields the best classification, while IMU sensors in the legs support better classification than IMU sensors in the arms.

**Figure 6.**
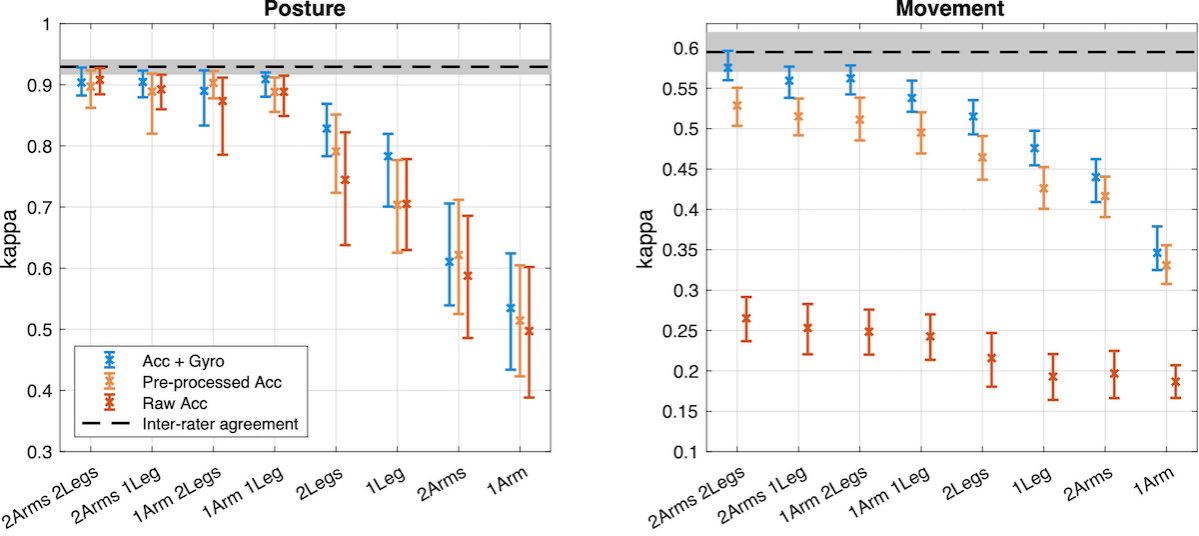
Effects of sensor modalities on the classifier performance. The findings extend the data presented in this figure by indicating sensor modalities by different colors. The 95% CIs of the performance values (kappa) are drawn with whiskers. The level of human inter-rater agreement (dashed line, 95% CIs depicted with gray) indicates the kappa levels needed to claim human-level accuracy. Please note the different y-axes for posture and movement to support within-task comparisons across configurations.

#### Sensor Modality

The effect of the sensor modality has the highest discrepancy between posture and movement: For posture, all 3 modalities perform rather acceptably, even though systematic differences can be observed (*t*_23_ range 2.2‐3.8; *P*=.035 to <.001). However, for movement, the performance of the “raw accelerometer” modality plummets (*t*_23_ range 28‐30; *P*<.001; 4 sensors from kappa=0.58 to kappa=0.27). In contrast, the pre-processed accelerometer modality manages to maintain performance in a more robust way when sensors are being reduced from the full setup, although the use of a gyroscope still results in superior performance (*t*_23_=14; *P*<.001).

An alternative representation of results is shown in Figure 6. Based on the above findings, the sampling frequencies could be pooled together, and the means of the kappa values and their 95% CIs (obtained by bootstrapping) are presented for recording configuration and modality. For posture detections, the classifier performance remains stable until the 1Arm1Leg sensor placement, and it deteriorates when only one limb type is recorded. For movement detections, however, the overall performance deteriorates gradually with all sensor placements. The CIs overlap for the 4- and 3-sensor placements when the recordings included both acc and gyro modalities. Notably, the reference configuration with acc and gyro modalities is nonoverlapping with all of the pre-processed accelerometer modality recordings.

#### Sampling Frequency

**Figure 7.**
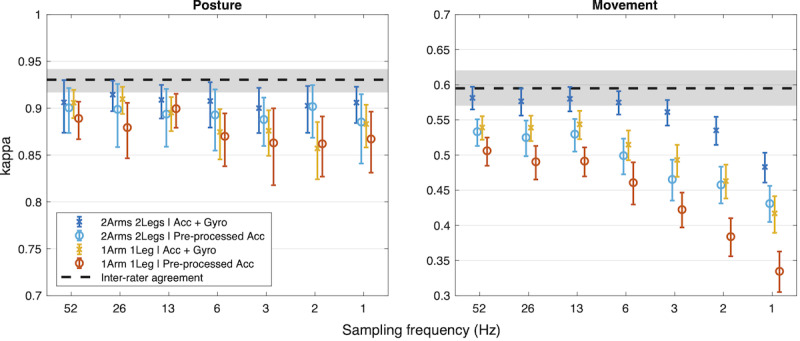
Effect of extreme sampling rate decimation on selected configurations. Notice the surprisingly high performance in posture detection for sampling rates as low as 1Hz. Please note the different y-axes for posture and movement to support within-task comparisons across configurations.

There were no systematic effects of sampling frequency either for posture or movement classifications within the supported range of the used Movesense sensors (13+ Hz). The median performance (kappa_pos_=0.85 and kappa_mov_=0.43) was slightly higher with 26 Hz, but there were no significant differences (*t*_23_ range 0.1‐1.1; *P*=.30-.90). Because the default sampling rate range did not present a differentiating effect for the posture and movement classification tasks, we conducted a further experiment to assess the effects of extreme downsampling with 4 candidate systems: 2Arms2Legs and 1Arm1Leg with either acc+gyro or pre-processed acc. The results are presented in Figure 7. Strikingly, the 2Arm2Leg system with a gyroscope retained unchanged performance down to a sampling rate of 6 Hz and was comparable to other full-sampled systems even at a 2 Hz sampling rate. We reason this to be the case due to 2 factors. First, even at a 1 Hz sampling rate, the raw accelerometer and gyroscope readings are, in fact, a good feature representation for orientation and movement. The accelerometer always includes the gravity vector, and the gyroscope reading is a good estimate of movement intensity. Second, the used neural network architecture takes consecutive frame-level features into account from a receptive field of 35 seconds, which significantly increases the amount of information available for each frame-level inference (ie, classification of single-frame features without surrounding context would drastically decrease performance).

### Experiment 2: Effect of Recording Configuration on Recording Level Category Distributions

Based on the result of experiment 1, we identified the following 4 recording configurations to be the most appealing in terms of maximizing some of the several measurement properties. First, (2Arm2Leg, acc and gyro, 13 Hz) as the best-performing system with the lowest supported sampling rate. Second, (1Arm1Leg, pre-processed acc, 13 Hz) as the best-performing 2-sensor configuration. Third and fourth, (1Arm and 1Leg, pre-processed acc, 13 Hz) as single-sensor configurations with large differences.

**Figure 8.**
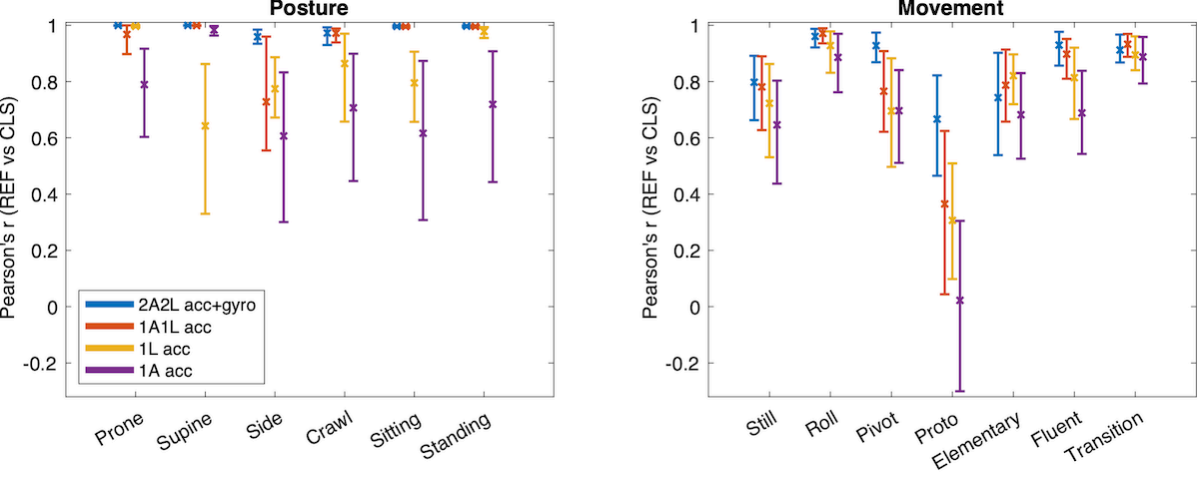
Effect of recording configuration on recording-level summary metrics. The correlations (Pearson *r*) between posture (left) and movement (right) distributions obtained using human annotation (REF) and those obtained from IMU recordings and classifier-based (CLS) detections.

Posture and movement category distributions provide a straightforward and explainable summary of the whole recording session. The results are presented in Figure 8. For the distributions of all posture categories, the reference recording configuration was very highly correlated to the distributions obtained from human expert annotations (range *r*=0.96-0.999; n=41). The sensor placement with 1Arm1Leg showed also a very high correlation (*r*=0.96-0.999) for all but the Side posture (*r*=0.73). The single-sensor recordings yielded a considerably lower performance: 1Leg has range (*r*=0.64-0.996), with most 4 of the categories having *r*<0.9, and 1Arm has the worst performance with range (*r*=0.61-0.94), with 5 categories having *r*<0.8.

The correlation between classifier-based and human expert-based movement category distributions was more variable, and there was also a more gradual deterioration of performance when compromising the recording configuration. Our reference configuration showed a range of 0.67-0.96, with 4 (out of 7) movement categories having *r*>0.9. For 1Arm1Leg the same metrics are in the range 0.34-0.97 (2 out of 7 categories with *r*>0.9). Overall, the movement categories “roll”, “fluent”, and “transition” seem to be the most robust in performance, whereas the “proto” category was least correlated to the human annotations.

### Experiment 3: Effect of Recording Configuration on Holistic Assessment of Motor Development

**Figure 9.**
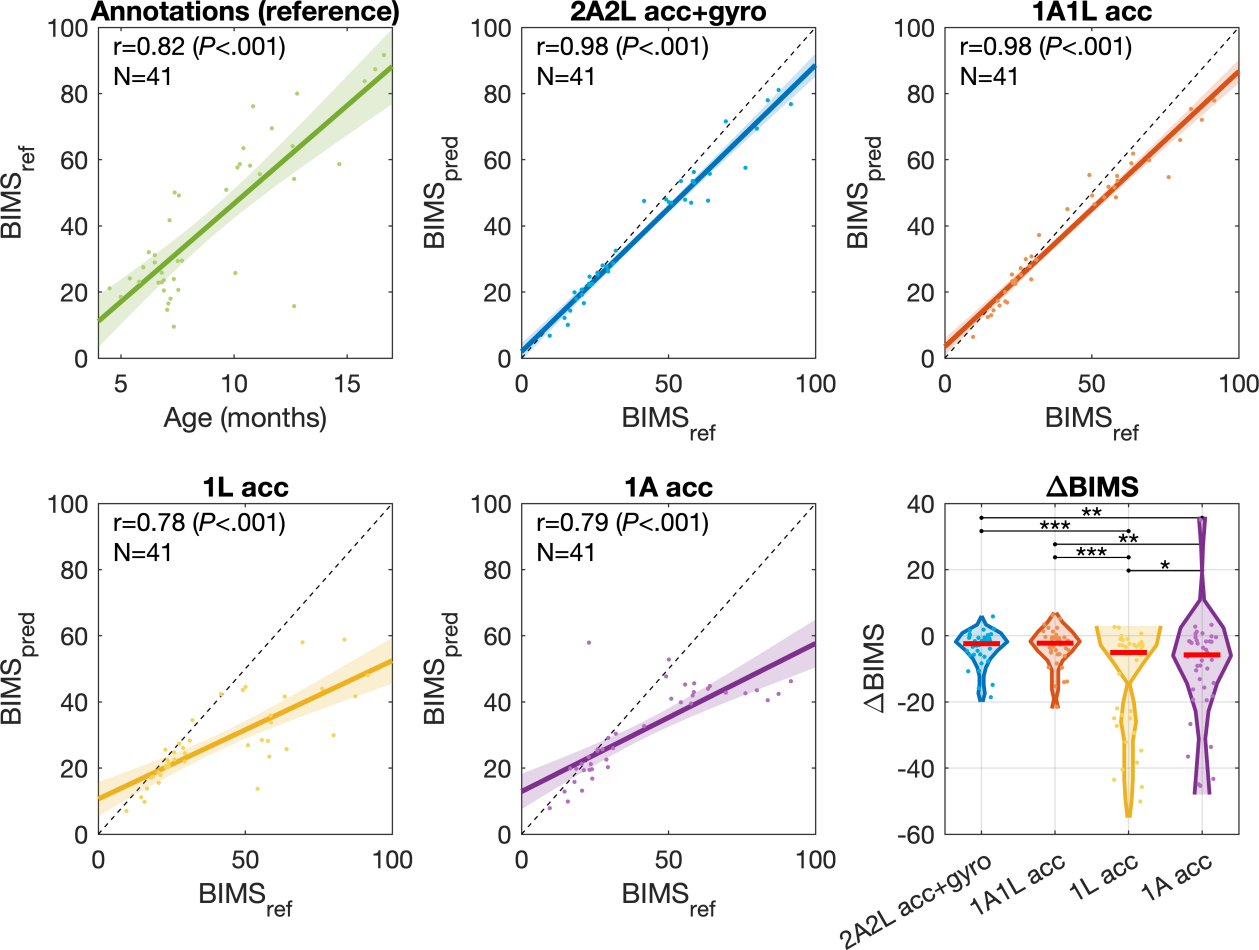
Effect of classifier-based inputs on the BIMS score (BIMS_pred_) compared with the reference (BIMS_ref_). Green: Scatter plot and correlation (Pearson *r*) of BIMS_ref_ compared with the chronological age. Blue, red, yellow, purple: Scatter plots and correlations of BIMS_pred_ compared with BIMS_ref_. Bottom right: Violin plots showing the ΔBIMS=BIMS_ref_ –BIMS_pred_ for the studied recording configurations. The red lines denote the median difference. Two-tailed paired *t* tests are used to test for the null hypothesis that the differences between systems’ performances are zero (*P<.05; **P<.005; ***P<.001). BIMS: BABA infant motor score.

Figure 9 presents the effect of reducing recording configurations to a holistic measure, here the scalar metric of a motor maturity score (BIMS). The first panel (green) indicates the reference score BIMS_ref_ that is obtained from the annotated distributions as a function of chronological age. The subsequent panels show the correlation of the IMU-recording-based and classifier-derived BIMS scores (BIMS_pred_) to BIMS_ref_. The dashed diagonal lines represent the ideal case where the scores are equal. The results show that the BIMS scores obtained from the 2Arm2Leg configuration are indistinguishable from those of 1Arm1Leg (*r*=0.98 for both), whereas the single-sensor configurations have clearly lower performance (*r*=0.78-0.79). Compared with the human expert annotations, the BIMS scores related to older age groups (higher baseline BIMS scores) are more difficult to predict, and this effect is most clearly seen in the 1-sensor variants (see bottom row of Figure 9): 2Arm2Leg median ΔBIMS=–2.4 (range –18 to 5.8), 1Arm1Leg –2.3 (range –21 to 6.8), 1Leg –5.0 (range –50 to 2.7), and 1Arm -5.7 (range –45 to 35). We assume this effect to be caused by the drastic qualitative shifts in gross motor abilities as the infants progress from lower (BIMS<50) to higher motor maturation (BIMS>50); this developmental time window is characterized by a shift from floor-based postures to increasingly upright postures and high postural variability.

### Summary of Results

Finally, Tables1  2 present the most relevant conclusions from the experiments: Sampling frequency did not have a significant effect on the classifier performance. Sensor modality did affect classifier performance, showing the best results with a combined accelerometer and gyroscope modality. However, a pre-processed accelerometer-only recording with separate low- and high-pass heads may produce reasonably good results with a minimalist measurement setup. For sensor placement, all 4- and 3-sensor configurations reach human equivalence in all measures of results. The best-performing 2-sensor configuration is obtained with a combined 1 arm and 1 leg placement, which has slightly lower-than-human equivalent performance in all other metrics except BIMS estimation. Finally, the one-sensor configurations perform poorly in all comparisons, while the proximal leg position is clearly better than an arm placement.

**Table 1.** The key takeaways of the experiments for sampling frequency and sensor modality for posture and movement classification. The effect of the sampling frequency is negligible. Ideally, both accelerometer and gyroscope are used, but relatively good performance can be reached with the pre-processed accelerometer system.

	Posture classification	Movement classification
Sampling frequency	52 ≈ 26 ≈ 13Hz	52 ≈ 26 ≈ 13 Hz
Sensor modality	Acc and gyro ≈ Pre-pr. acc ≈ Raw acc	Acc and gyro ≳ Pre-pr. acc >> Rawacc

**Table 2.** The key takeaways of the experiments for sensor placement at various levels of analysis. The minimal system is the variant with 1Arm1Leg sensor placement, 13 Hz sampling rate, and pre-processed accelerometer modality. HEQ: human equivalence in terms of inter-rater agreement.

Sensor placement	High-resolution		Overall statistics		Holistic
	Posture	Movement	Posture	Movement	BIMS
4 (2A2L)	HEQ	HEQ	HEQ	HEQ	HEQ
3 (1A2L or 2A1L)	HEQ	HEQ	HEQ	HEQ	HEQ
2 (1A1L)	↓	↓	(↓)	↓	HEQ
1 (1L)	↓↓↓	↓↓↓	↓↓↓	↓↓↓	↓↓↓↓
1 (1A)	↓↓↓↓	↓↓↓↓	↓↓↓↓	↓↓↓	↓↓↓↓

## Discussion

### Principal Results

#### Overview

This study shows that reductions in the IMU recording configurations lead to systemic and predictable compromise at all analytical levels, ranging from high temporal resolution analyses to recording-level statistics and a holistic assessment of motor performance. (Tables1  2). While the present dataset is collected from young infants during their spontaneous activity, the studied movement behaviors [28] present a natural repertoire of well-defined movements and postures at any age. We, therefore, posit that the findings on high-resolution posture and movement detections are generalizable to other age groups as well, though the lower-resolution movement behavior in the older participants is more structured and predictable. Thus, the highly variable infant data provides a stress test for the overall methodology. Most importantly, (1) sensor placement is critical and at least one lower and one upper extremity sensor is essential for all high-resolution detections of posture or movement categories, (2) gyroscope data is significant for classifying movement types, and (3) data sampling rate has no meaningful effect on analyses.

When the analyses are based on complex pipelines, such as machine learning-based classifiers, the links between compromises in data versus results escape heuristic predictions. Instead, direct experimental evidence is needed, preferably in the context of an authentic end-to-end solution, such as the MAIJU recordings for infants’ motor assessment used in our work. The strong effects of recording configuration, in particular the very low analytic accuracy of single sensor recordings, may seemingly conflict with the long history of actigraphy recordings [13,17,20]. Our study targeted high-fidelity detections of qualitatively different postures and movements, whereas actigraphy analyses measure the intensity (or amount) of movements to be categorized into different activity levels, such as sedentary, light, or moderate to vigorous activities [15,16]. Our present work does not aim to claim a universal superiority of any recording configurations. Instead, our findings emphasize the tight interplay, or trade-off, between the chosen recording configurations and the attainable analyses and study questions. Another potential comparison is the range of recently launched sport and wellness wearables that use one sensor to provide the user with logs of selected activity types, such as running, walking, or cycling. The apparently successful performance of those applications is based on using very long time-epochs (minutes to tens of minutes) and analyzing only monotonous and rhythmic movement patterns. Our results from human video annotations confirmed that the behaviorally relevant time resolution needs to be in seconds rather than minutes, and a significant part of the natural movement and posture categories of daily activity are not monotonous or rhythmic, at least in the studied infant population. While the present results and the presently studied movement measures are directly generalizable to any age group, it is important to note that studies using other movement categories (ie, other analysis targets) would benefit from their respective trade-off analyses.

The main purpose of reducing the recording configuration is to simplify movement studies for wider scalability and easier analytics:

#### Sampling Frequency and Data Rate

We show that the data rate can be reduced without loss of analytic gain from the original setup to 13 Hz, 2-sensor acc only recordings, corresponding to a compression factor of 16 (from 1248 numbers per second; 5.0 kilobytes/s with 32-bit floats to 78 numbers per second; 0.4 kilobytes/s). Furthermore, our results suggest that an even more radical reduction of the sampling rate to 2‐6 Hz is feasible, especially if a higher number of sensors can be maintained. This is a novel finding that might have significant ramifications for future research directions, however, the current findings are directly applicable to the specific movement and posture categories used in our study pipeline. Other HAR classification targets with a wider dynamic range of physical movements may be more sensitive to lowering the sampling rate. Lower sampling rates improve the pipeline at all levels: First, smaller IMU data files per time enable storage of longer recordings in the sensor memory [39]. Compared with real-time data streaming, local storage is easier for the user, and it yields substantial savings on the sensor battery (–96.8 μA) [40]. Second, the transmission of the recorded data over a wireless connection becomes much faster, and in the case of continuous streaming, it improves the stability of the wireless connection (here Bluetooth 5.0 low-energy was used).

#### Sensor Modalities

Reduction in sensor modalities to accelerometer only has direct effects on power consumption because gyroscopes consume considerably more power than accelerometers (approximately 5 times in the used Movesense sensor; 46 μA for 13 Hz acc, 227 μA for 52 Hz acc+gyro [40]). Additionally, dropping the gyroscope will also halve the data rate.

#### Sensor Number and Placement

Reduction in the number of sensors without significant effects on analysis performance gives 2 kinds of advantages: First, data recorded with initially higher sensor numbers can be used even in the event of sensor dropouts, either due to an actual recording failure of individual sensors or due to rejection by quality control afterward. Second, a lower number of sensors will also imply cheaper construction and easier use of the wearables. Here, we showed that a 2-sensor configuration is feasible in most situations if the data is reliably collected from at least one upper and one lower limb; yet, additional sensors are always improving the robustness by providing redundancy in case of technical failures that may be unpredictable in real-world environments.

### Limitations

While our results contain readily translatable interpretations to wider use cases, the exact experimental design implies some limitations. First, our reference IMU data were obtained using a recording setup that was previously optimized for infant studies. Other anatomic locations (eg, distal limbs, trunk, or head [41,42]) could add further insights for use cases outside of the infant gross motor assessment. Our own unpublished studies indicated that distal sensor placements would not improve analytic reliability enough to justify the extra practical challenges coming from adding distal IMU sensors in wearable suits. Additionally, distal placements would come at the cost of far higher movement complexity in most activities. Regarding trunk and hip placement, our data does not inform it, but the lateral proximal sensor location in the leg comes close to hip placement.

Second, our work focused on posture and movement classifications of infants from the age they begin moving until they walk fluently. The apparently canonical postures and movements used in our classifier task [29] are likely applicable to any age group, but other groups were not explicitly assessed. However, we posit that naturalistic IMU datasets from adults including substantial sedentary behavior could well lead to better performance with more reduced recordings [27]. Nevertheless, we suggest that our present results and the conclusions thereof should be taken primarily as recommendations for a “safely sufficient” recording setup for the universally applicable posture and movement categorization task. Finally, it is important to note that our classifiers did not extend to more subtle movement characteristics, such as symmetry or synchrony between limbs, which may be important in the medical context [43]. Such analyses, however, require symmetrical sensor placements and preferably high sampling rates, such as our 4-sensor reference configuration.

### Comparison With Prior Work

Our findings are fully in line with a larger body of prior literature (both for infants [6,28] and for adults [41,42,44]) and with the intuitive reasoning that reductions in the recorded data would compromise the analytically extractable information. However, we are not aware of prior studies performing so an extensive and systematic study, including so widely ranging recording configurations and diverse analytical outputs, where data collection has been based on naturalistic observation of behavior. Our results need to be compared with prior work separately for each study dimension. Regarding sensor number and placement, our findings are fully compatible with prior studies showing that 3 to 4 sensors in infants [4,6,28], or at least 2 sensors in adults [41,42], may provide sufficient analysis of postures at lower time resolutions. Our findings extend prior work by showing that 2-arm or 2-leg only setups are not adequate. We also extend our analysis outputs to movement classification, where the gyroscope supports clearly better analytic accuracy. Regarding the resolutions of our movement classifier, our study extends significantly the prior works by using a very high temporal resolution as well as discrimination between widely varying movement types [29]. Our algorithms operate at the second-by-second level for both posture and movements, whereas prior studies have provided multisecond [4,6], or more often multiple-minute resolution [45], or even coarser than that in many consumer electronic applications. It is clear that averaging movement data over longer time windows will improve classification, but this comes at the cost of losing the natural behavioral variability. Here, we showed empirically using video-based annotations that natural behavior is characterized by altering postures and movements at the level of seconds. Thus, an ecologically valid and informative movement analysis must also work at the same time resolution. It is striking in this context that most of the published HAR research has used “acted out” data collection, where the classifiers are trained (and validated) using movement recordings where participants’ performance is instructed (eg, [4,17,42,46,47]). Such classifiers may perform seemingly well, but they grossly simplify the naturalistic human movement activity behavior, leading to significant concerns about the lack of real-world validity and applicability when using the common recording configurations and analytic approaches [27]. The need for higher quality validation protocols, improved gold-standard reference methods (eg, video), and more realistically assigned classification targets [29] is imperative when the movement recordings are applied to assessing real-world behavior using wearable systems. A recent thorough review by Giurgiu et al [27] concludes with the statement (sic): “Future research should strongly aim at biological state and posture or activity outcomes and strive for standardized protocols embedded in a validation framework”, which is precisely addressed by this article.

### Conclusions

This study identified the minimalist setup for adequate posture and movement tracking as: at least a combination of one upper and one lower extremity sensor, 13 Hz sampling frequency, and at least an accelerometer, but preferably also a gyroscope. However, optimizing recording configurations always comes at a price, and striking the balance between gains and losses must be done with respect to each analysis target and the study question. For instance, a posture-based behavioral assessment may benefit from a very long recording time, even up to a week, supporting the use of the minimalist setup. However, thorough characterization of gross motor performance needs high accuracy in both posture and movement measures, while it does not benefit from excessively long recording times [29,30]. For the particular purpose of infant recordings with optimal analytic gain, it is recommended to use the full reference configuration (as in the MAIJU wearable setup [29]), including 4 sensors with both accelerometer and gyroscope modalities. The systematic assessment of analytic compromises provides future IMU sensor users with an evidence-based guide to optimize their recording configurations for each particular use case.

## Abbreviations

BIMS: BABA infant motor score
HAR: human activity recognition
IMU: inertial measurement unit
MAIJU: motor ability assessment of infants with a jumpsuit
